# Editorial Perspective: Issues for DSM 6 – an Alternative Model for Neurodevelopmental Disorders to enhance nosological validity and clinical utility

**DOI:** 10.1111/jcpp.70017

**Published:** 2025-08-04

**Authors:** Michele Poletti, Antonio Preti, Andrea Raballo

**Affiliations:** ^1^ Department of Mental Health and Pathological Addiction, Child and Adolescent Neuropsychiatry Service Azienda USL‐IRCCS di Reggio Emilia Reggio Emilia Italy; ^2^ Department of Neuroscience University of Turin Turin Italy; ^3^ Chair of Psychiatry Faculty of Biomedical Sciences, University of Southern Switzerland Lugano Switzerland; ^4^ Cantonal Sociopsychiatric Organisation Mendrisio Switzerland

**Keywords:** Neurodevelopmental disorders, alternative dimensional model, severity levels, phenotypes, dimensional profiles.

## Abstract

The categorical diagnostic approach in the DSM‐5 for Neurodevelopmental Disorders often reveals significant limitations, as high rates of comorbidity are common across conditions such as ADHD, autism spectrum disorder, and developmental coordination disorder. This co‐occurrence aligns with a neuroconstructive dimensional perspective of neurodevelopment, which emphasizes the interconnectedness of cognitive, motor, and social impairments evolving throughout development. This perspective challenges modular and categorical views of neurodevelopmental phenotypic manifestations. Envisioning the DSM‐6, a proposed dimensional Alternative Model for Neurodevelopmental Disorders (AMND) could integrate functioning severity and pathological traits, drawing inspiration from DSM‐5 innovations, such as the Autism Spectrum Disorder severity levels. Such a model could facilitate nuanced profiling of individual strengths, needs, and developmental risks, accommodating both categorical and dimensional diagnostic approaches. This framework could also improve early identification of vulnerabilities to severe mental illnesses and clarify the developmental antecedents of adult‐onset psychiatric conditions, offering pragmatic insights for clinical interventions and prognosis.

Accurate diagnosis and classification of mental disorders are vital for building a robust and effective healthcare system driving advancements in research. Historically, however, psychiatric nosology was considered a less dynamic field, with early 20th‐century classification systems developed primarily for administrative purposes rather than clinical guidance. This changed dramatically with the publication of DSM‐III in 1980, which marked a pivotal moment in making psychiatric classification a central focus of mental health research and practice. Since then, the various iterations of the DSM have had a substantial impact in the field, partly incorporating descriptive features that go beyond the canonical categorical approach. For example, the DSM‐5 introduced a simplified and pragmatic dimensional framework to personality disorders, that is, the Alternative Model for Personality Disorders (AMPD), while maintaining the overall categorical architecture adopted in previous versions.

This development sought to overcome the well‐known limitations of a purely categorical approach to personality disorders, which frequently leads to the co‐occurrence of different disorders, often (although not exclusively) within the same personality cluster, and is associated with low stability over time (García, Gutiérrez, García, & Aluja, 2024). Overall, the AMPD assesses personality pathology in terms of both the dimensional severity of personality dysfunction (Criterion A) and the presence of pathological personality traits (Criterion B), spanning across five domains (negative affectivity, detachment, antagonism, disinhibition, psychoticism) and 25 lower‐order facets.

## A dimensional approach to neurodevelopmental disorders

A similar set of limitations, inherent to a purely categorical approach, can be observed in the diagnostic categories within the DSM‐5 section on Neurodevelopmental Disorders (i.e., Intellectual Disabilities, Communication Disorders, Autism Spectrum Disorder, Attention Deficit/Hyperactivity Disorder, Specific Learning Disorder, and Motor Disorders) where co‐occurrence or comorbidity is more the rule than the exception (Dewey, 2018). For example, the majority of children with Developmental Coordination Disorder meet criteria for at least one other neurodevelopmental condition, such as ADHD, dyslexia, specific language impairments, or autism spectrum disorder. Nearly half of children with ADHD exhibit movement challenges that align with a Developmental Coordination Disorder diagnosis, and 20%–30% have tic disorders. High comorbidity rates are also noted among dyslexia and dyscalculia (28%–64%), as well as between dyslexia and ADHD (10%–40%) and between dyscalculia and ADHD (12%–36%). Neurodevelopmental co‐occurrences are common among children with ASD as well, including ADHD symptoms (30%–50%) and motor symptoms in the majority of cases.

This categorical co‐occurrence, along with the associated widespread comorbidity, aligns with long‐standing conceptualizations of altered neurodevelopment as a foundation for many phenotypic manifestations during childhood and the critical periods of development. While this remained largely a speculative hypothesis for decades (Schmitt, 1975), empirical evidence at both the neural and genetic levels, including polygenic overlap, has now accumulated to support this perspective (Moreno‐De‐Luca et al., 2013; Parenti, Rabaneda, Schoen, & Novarino, 2020). Moreover, the co‐occurrence of neurodevelopmental disorders is consistent with a neuroconstructivist approach, which focuses on the ontogenesis of cognitive functions and their intertwined co‐development due to reciprocal cascading effects in both typical and atypical development (D'Souza & Karmiloff‐Smith, 2017). Indeed, from a clinical and psychopathogenetic viewpoint, the phenotypic isolation of a neurodevelopmental disorder from other neurodevelopmental disorders could only be conceived under a purely modular perspective, where one or more cognitive modules are impaired and the rest of the neurocognitive system remains unaffected. However, empirical data do not support this view (Dewey, 2018).

In the current era of rethinking psychiatric diagnoses, diagnostic systems, and psychopathological conceptualizations [see, e.g., the Research Domain Criteria (RDoC) and the Hierarchical Taxonomy of Psychopathology (HiTOP)] (Michelini, Palumbo, DeYoung, Latzman, & Kotov, 2021), neurodevelopment has garnered increasing attention as an independent psychopathological spectrum (Michelini et al., 2024), which complements the internalizing, externalizing, and psychotic spectra and may serve as an early organizing framework through which understanding both neurodevelopmental disorders according to DSM‐5 and the premorbid development of vulnerability to later, severe mental illness (Raballo, Poletti, & Preti, 2024). For instance, applying a neurodevelopmental perspective to schizophrenia suggests that the prodromal emergence of the first attenuated psychotic symptoms during adolescence or early adulthood represents merely the visible tip of an iceberg. What lies beneath, though less visible, is more substantial, including the gradual accumulation of delays and difficulties in cognitive, motor, and social‐interpersonal domains, their interaction, and their cumulative effect, starting in childhood and spanning the developmental years (Raballo et al., 2024).

Overall, a dimensional approach to psychological problems, including neurodevelopmental phenotypic manifestations, may offer significant advancements compared to a categorical approach (Lahey, Tiemeier, & Krueger, 2022):

- Greater adherence to empirical assessment (Haslam, McGrath, Viechtbauer, & Kuppens, 2020).
- Increased reliability.
- Improved prognostic precision regarding outcomes.
- A more respectful and comprehensive consideration of individual needs.
- Reduced risk of reifying psychological problems.
- Better suitability for a dynamic understanding over time (and throughout development).
- Reduced stigma.

## Alternative model for neurodevelopmental disorders

Therefore, drawing on the empirical and clinical advancements offered by a dimensional approach that complements rather than replaces a categorical framework, especially in a field marked by high comorbidity among partially overlapping conditions (such as the AMPD for personality disorders), a substantial innovative step toward DSM 6 could be developing a dimensional *Alternative Model for Neurodevelopmental Disorders* (AMND). In line with the diagnostic criteria of the AMPD, a neurodevelopmental disorder could be dimensionally diagnosed according to the AMND in terms of both the dimensional severity of functioning (Criterion A) and the presence of pathological neurocognitive and behavioral traits (Criterion B). A challenging task could be the characterization of the severity of functioning (putative Criterion A). Indeed, while in the AMPD, personality functioning is described according to four elements (identity, self‐direction, empathy, and intimacy), adapting such criterion to AMND would require an anchoring to the varying degrees of independence, initiative, social interaction, and overall well‐being according to age‐related expectancies, for example, as they are incorporated in the Children's Global Assessment Scale (CGAS; Shaffer et al., 1983). Otherwise, capitalizing on the infra‐category dimensional scoring of severity introduced by DSM 5 for Autism Spectrum Disorder, in AMND, the Criterion A should be anchored to needs of support [Level 1 (‘requiring support’), Level 2 (‘requiring substantial support’), and Level 3 (‘requiring very substantial support’)].

The clinical characterization of the descriptive traits of AMND could capitalize on the core clinical characteristics inherent to the diagnostic criteria of distinct categories of neurodevelopmental disorders, establishing connections between specific deficits and their corresponding neurocognitive functions (see Table 1). Such clinical characterization would facilitate the description of phenotypes along dimensional neurodevelopmental profiles that encompass various neurocognitive domains. This approach would also allow for categorical diagnoses when certain symptoms are hierarchically salient, highlighting the severity of specific traits over others. This broader profiling could provide a more comprehensive and representative picture of individual strengths and weaknesses, not limited to the most phenotypically evident trait of impairment. Furthermore, this would facilitate a clearer identification of needs and areas for proximal development, compared to what categorical diagnoses currently allow. Moreover, such a neurodevelopmental profile could be pragmatically useful for the clinical characterization and timely inception of the broad range of at‐risk psycho‐behavioral phenotypes associated with early‐onset psychiatric disorders, such as very early‐onset schizophrenia, pediatric bipolar disorder, or pediatric obsessive‐compulsive disorder. Similarly, such a neurodevelopmental profile could be central for a retrospective detection of premorbid vulnerabilities or antecedents of adult‐onset psychiatric disorders (Raballo et al., 2024).

**Table 1:** Potential traits and dimensions for profiling neurodevelopmental phenotypes

Trait	Dimension	Phenotypic expression of altered neurodevelopment	Possible corresponding categorical diagnoses based on hierarchical primacy of the domain
Motor	Gross‐motor functions	Dyscoordination	Developmental coordination disorder
	Aiming and catching		
	Fine motor functions		
Communication	Receptive language	Receptive language impairment	Language disorder
	Expressive language	Expressive language impairment	
	Social use of verbal and non verbal communication	Pragmatic impairment	Socio‐communicative disorder
Autistic	Social interaction	Deficits in social interactions	Autism spectrum disorder
	Behavior, interests, or activities	Restricted, ripetitive	
Intellectual	Abstract reasoning	Intellectual weakness or disability	Intellectual disability
	Verbal comprehension		
Executive functions	Working memory	Executive impairment	
	Set‐Shifting		
	Inihibition		
	Planning		
Attention	Attention	Disattention	Attention deficit/Hyperactivity disorder
	Activity	Hyperactivity/impulsivity	
Learning	Reading	Learning impairment	Learning disorder
	Writing		
	Handwriting		
	Math		

From a dimensional perspective, some key testable predictions could be delineated:

- Greater severity of symptomatic impairment in one trait is associated with an increased likelihood of impairments in other ontogenetically interdependent traits (*cross‐dimensional severity*).
- Greater severity of symptomatic impairment in one or multiple neurocognitive traits is associated with an increased likelihood of co‐occurring psychopathological symptoms (whether along the internalizing and/or externalizing spectrum or within thought processes) (*psychopathological expressivity*).
- The greater the probability of co‐occurring neurocognitive and psychopathological symptoms in infancy and childhood (i.e., psychopathological expressivity), the less likely it is that pubertal development will alleviate or compensate for these symptoms (*prognostic persistence*).

These indexes of severity (cross‐dimensional severity, psychopathological expressivity, prognostic persistence) may contribute to the emergence of later‐onset mental disorders and increase the likelihood of later transitions toward other mental disorder diagnostic categories in adulthood.

## Conclusions

Overall, incorporating a dimensional diagnostic system for neurodevelopmental disorders (the AMND) into DSM‐6 could be a pragmatically straightforward yet heuristically powerful transformative innovation. In this context, the DSM‐5 Alternative Model for Personality Disorders (AMPD) serves as both a valuable example and a conceptual framework. While it is important to recognize that the simplicity of categorical approaches has historically provided clinicians with a clear and accessible vocabulary for discussing mental health issues, remaining a practical guide for complex clinical decision making (Sonuga‐Barke, 2022), the transition toward a concurrent alternative dimensional model for neurodevelopmental disorders promises to enhance diagnostic precision and clinical utility. By embracing this approach, we can better capture the complexities of neurodevelopmental conditions, enhance early risk stratification for future conditions, and improve personalized treatment and outcomes for individuals affected by them.

## Data Availability

Data sharing not applicable to this article, as no datasets were generated or analyzed during the current study.
